# Joint Modeling of 5G Slicing Reliability and Frequency Regulation Revenue for Virtual Power Plants: A Stackelberg Game Approach

**DOI:** 10.3390/s26154735

**Published:** 2026-07-26

**Authors:** Xianing Jin, Menghan Zhu, Pei Liu, Xin Liu, Shigong Jiang

**Affiliations:** 1 State Grid Economic and Technological Research Institute Co., Ltd., Beijing 102209, Chinajiangshigong@chinasperi.sgcc.com.cn (S.J.); 2 Beijing Key Laboratory of Low-Carbon and Flexible Urban Power Supply Technology and Equipment, Beijing 102209, China; 3 College of Mathematics and Physics, Beijing University of Chemical Technology, Beijing 100029, China; 4 College of Mechanical and Electrical Engineering, Beijing University of Chemical Technology, Beijing 100029, China; 5 Department of Computer Science and Technology, Tsinghua University, Beijing 100084, China; liuxin1998@mail.tsinghua.edu.cn

**Keywords:** frequency regulation response, virtual power plant, 5G slicing, frequency regulation revenue, resource pricing, Stackelberg game

## Abstract

Virtual power plants (VPPs) are emerging as flexible resources for automatic generation control (AGC) frequency regulation by coordinating geographically dispersed distributed energy resources. However, the timely execution of AGC commands is highly sensitive to communication latency and reliability, and conventional cellular networks may fail to provide stable service guarantees under high concurrency regulation scenarios. To address these issues, this paper proposes 5G radio access network (RAN) slicing technology to provide dedicated communication resources for VPP frequency regulation command transmission. First, the resulting communication performance is further embedded into the AGC performance score, establishing an explicit mapping from network slicing resources to VPP regulation revenue. Next, a Stackelberg game model between the telecom operator and the VPP is constructed to achieve coordinated optimization of network slice resource pricing and allocation. Simulation results show that the proposed method can significantly improve AGC command transmission reliability and frequency regulation tracking performance, while achieving a coordinated enhancement of both VPP regulation profit and telecom operator revenue.

## 1. Introduction

Against the backdrop of the global energy transition, the large scale integration of intermittent renewable energy sources such as wind and solar power has become a key trend in the development of electricity systems. Policy documents such as the EU Fit for 55 package [1] and the U.S. Inflation Reduction Act [2] further confirm this direction of transition at the policy level. At the same time, distributed energy resources such as rooftop solar, battery storage, and electric vehicles are seeing unprecedented levels of adoption. VPPs utilise big data analytics, artificial intelligence, and advanced information and communication technologies to aggregate and coordinate geographically dispersed renewable energy sources, energy storage systems, flexible loads, and demand response assets. By aggregating resources such as distributed generation, energy storage, and controllable loads, and relying on a unified monitoring, control, and optimised dispatch mechanism, VPPs achieve coordinated regulation of resources on both the generation and consumption sides [3,4]. In frequency regulation services, VPPs receive Automatic Generation Control (AGC) commands issued by the dispatch centre to coordinate the control of aggregated distributed resources, thereby enabling a rapid response to grid frequency deviations and enhancing system frequency regulation capability and operational flexibility.

**Figure 1:**
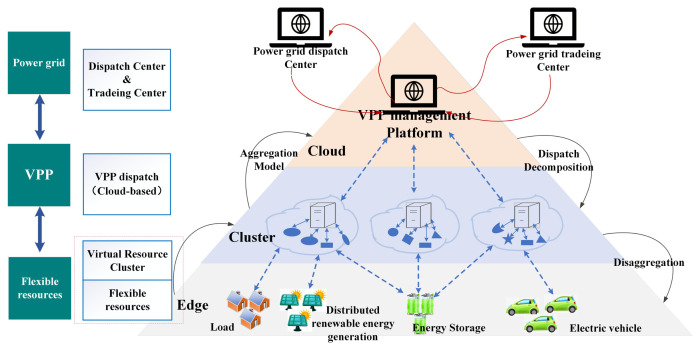
VPP architecture.

Typically, VPPs mitigate dynamic fluctuations in the power system through the aggregation and coordinated dispatch of distributed energy resources. These vast distributed resources rely on secure and reliable communication networks to enable high frequency data collection and the generation of real time monitoring data [5,6]. The VPP network architecture is shown in Figure 1. However, with the widespread deployment of distributed energy resources, including photovoltaics, wind turbines, energy storage systems, and controllable loads, VPPs have expanded rapidly in scale. Consequently, their underlying communication networks exhibit characteristics such as massive terminal counts, high service concurrency, extensive geographic coverage, and pronounced load fluctuations [7], which in turn have precipitated a substantial surge in the volume of data gathered and uploaded by these terminals [8]. At the same time, VPP frequency regulation control requires the real time transmission of AGC commands to various distributed resources via the communication network, as well as the reception of their operational status information to complete closed loop control. When the communication network experiences increased latency or data packet loss, frequency regulation commands may fail to be transmitted to resource units in a timely or accurate manner, leading to delayed frequency regulation responses or increased tracking errors, which ultimately affect the VPP’s frequency regulation performance and market revenue. Consequently, as the scale of VPPs continues to expand, higher demands are placed on advanced communication network architectures and communication technologies [9,10,11]. Therefore, there is an urgent need to establish an analytical relationship between communication performance and frequency regulation response, and to further achieve coordinated optimization between power systems and communication networks in order to elucidate the mechanisms by which communication network performance affects VPP frequency regulation performance.

Studies on VPPs and aggregated distributed resources participating in power system frequency regulation have mainly focused on resource aggregation modeling, frequency regulation control strategies, and market based operation mechanisms. Han et al. [12] developed an optimization model for electric vehicle aggregators providing frequency regulation services and demonstrated the feasibility of aggregated electric vehicles participating in frequency regulation ancillary services. Liu et al. [13] further considered the state of charge preferences of electric vehicle users and proposed a scheduling and control method for electric vehicles to assist in frequency regulation. At the VPP level, Adabi and Marinescu [14] proposed a strategy for dynamic VPPs to participate directly in secondary frequency control. Guo et al. [15] investigated control methods for VPPs to support frequency regulation in the main grid and showed that VPPs can enhance system frequency response by coordinating distributed generation, energy storage, and controllable loads. These studies indicate that VPPs have the technical potential to participate in frequency regulation. However, the communication transmission process and its impact on frequency regulation response are generally simplified.

The impact of communication network performance on the participation of distributed resources and VPPs in frequency regulation has also received attention. Ko and Sung [16] investigated the effect of cellular communication delays on electric vehicle aggregators providing frequency regulation services and showed that communication delays can impair the response of aggregated resources to regulation commands. Ledva et al. [17] analyzed the control errors caused by communication delays and modeling errors when demand response resources participate in frequency regulation. Hui et al. [18] established a modeling and control method for flexible load frequency regulation services considering communication delays and detection error compensation. In the VPP context, Cao et al. [19] investigated the effects of channel noise and time varying topology on the economic dispatch performance of VPPs under nonideal communication networks. Zhou et al. [20] incorporated communication spectrum allocation into the coordinated optimization of electric vehicle VPPs. Feng et al. [4] studied VPP frequency regulation service provision based on 5G RAN slicing and linked communication resource allocation with frequency regulation service performance. These studies show that communication delay, transmission reliability, and communication resource allocation affect the frequency regulation response of aggregated resources or VPPs and may further influence their market revenue. However, most existing research has been conducted from the perspective of communication constraints or frequency regulation control. The quantitative relationship among 5G slice resource allocation, delay violation probability, AGC command transmission success rate, and VPP frequency regulation tracking performance remains insufficiently characterized.

With the development of fifth generation mobile communication technology, 5G network slicing provides a promising communication solution for critical control services in power systems. 5G RAN slicing enables multiple logically isolated virtual networks to be created over the same physical infrastructure, allowing customized resource allocation and QoS guarantees for different service types [11,21]. For VPP frequency regulation services, 5G slicing can provide dedicated communication resources for AGC command transmission, thereby reducing communication latency and packet loss and improving the real time performance and reliability of frequency regulation control. In recent years, considerable research has been conducted on the application of 5G technology in smart grid services [22]. The study in [23] shows that RAN slicing can support smart grid self healing services by dynamically managing communication resources under stringent service requirements. Feng et al. employed 5G radio access network (RAN) slicing to separate frequency regulation command traffic from other service traffic, thereby mitigating external interference in the transmission of distributed resource commands and improving the reliability of VPP frequency regulation services [4]. These studies demonstrate that 5G communication technology can provide efficient, secure, and reliable communication support for critical smart grid services. However, most existing studies assume that communication service providers can offer sufficient 5G communication resources to VPPs. The resource constraints faced by communication service providers in practical operation, as well as their economic incentives for participating in power system services, have not been fully considered.

As an increasing number of third party aggregators become involved in the operational control and market trading of VPPs, the communication architecture of the power system is gradually evolving from traditional dedicated communication networks towards a multi network convergence architecture. The deep integration of communication networks with the power system has also become a key direction for the development of smart grids [24]. In scenarios where VPPs participate in frequency regulation, they need to obtain network slice resources from telecommunications operators to ensure the reliable transmission of Automatic Generation Control (AGC) commands and to improve their accuracy score (AS) in the frequency regulation market. However, the procurement of network slice resources increases the operational costs of VPPs, while communication service providers must also balance network resource utilization and operational revenue when providing slice services. Consequently, how to achieve reasonable pricing and optimised allocation of network slice resources while meeting the frequency regulation performance requirements of VPPs is a critical issue that urgently needs to be addressed in the coordinated operation of power systems and communication networks.

To address the aforementioned issues, this paper first applies effective capacity (EC) theory to characterize the delay violation probability of AGC command transmission as a function of the subscribed 5G slice resources. The delay violation probability is then mapped to the AGC command transmission success rate and the VPP frequency regulation performance score. On this basis, a Stackelberg game is formulated between the communication service provider and the VPP, where the communication service provider determines the unit slice price and the VPP determines the number of subscribed slices. The equilibrium strategies are derived by backward induction, enabling joint analysis of slice pricing, slice subscription, communication reliability, and VPP frequency regulation performance. The primary innovations of this paper can be summarized as follows:

- Developed an effective capacity model for 5G slicing enabled VPP frequency regulation, mapping slice resource allocation to delay violation probability and AGC command transmission success rate, and quantifying the impact of communication reliability on VPP AGC tracking performance.
- Proposed a communication aware revenue model for VPP frequency regulation by incorporating the AGC performance score and slice cost into the VPP profit function, quantifying the tradeoff between communication resource cost and frequency regulation revenue.
- Formulated a Stackelberg game framework for slice pricing and subscription in VPP frequency regulation, where the communication service provider sets the unit slice price and the VPP determines the number of subscribed slices by balancing regulation benefit and communication cost. Derived the equilibrium pricing and subscription strategies for both participants.

The remainder of this paper is structured as follows: Section 2 establishes the system model; Section 3 presents problem solving and analysis; Section 4 conducts simulation analysis; and Section 5 draws conclusions.

## 2. Systems Model

### 2.1. Network Model

**Figure 2:**
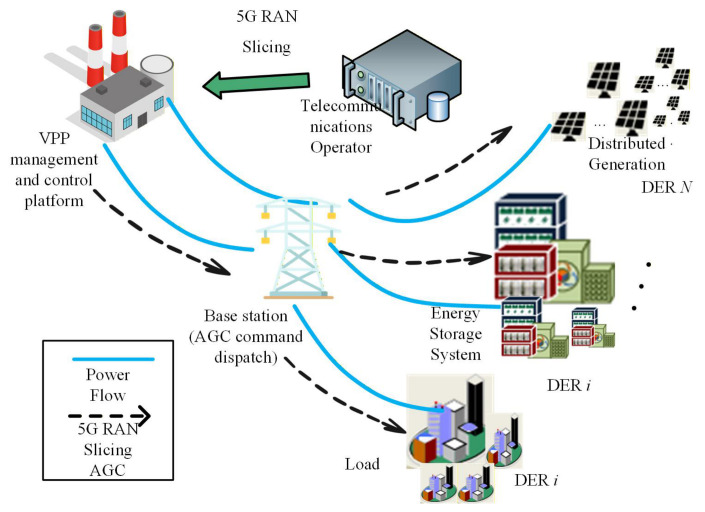
System Model.

As shown in Figure 1, the proposed system architecture adopts a cyber physical framework wherein DERs, including distributed solar power production, power storage systems, and controllable loads are geographically aggregated into a VPP via the distribution network. As illustrated in Figure 2, the physical layer is characterized by bidirectional power flows (denoted by solid blue lines) interconnecting generation, storage, and consumption entities, thereby establishing the electrical coupling required for energy exchange among dispersed units.

The cyber layer leverages 5G RAN slicing orchestrated by a telecommunication operator to provision deterministic, ultrareliable, and low latency communication channels for real time VPP dispatch. Specifically, the VPP Management and Control Platform exchanges AGC command signals with individual DER units through dedicated network slices via base stations (BS), effectively decoupling the information flow from the underlying electrical power transfer. This sliced communication infrastructure enables the VPP aggregator to acquire heterogeneous operational states, compute aggregated regulation setpoints, and disseminate power commands to DER *i* in a closed loop coordinated manner, allowing the ensemble of distributed resources to collectively deliver grid auxiliary services, such as power frequency regulation and active load balancing.

### 2.2. Frequency Regulation Model for Distributed Energy Resources

VPP aggregated distributed energy resources typically include distributed energy storage systems and controllable loads. Although these two types of resources have different regulation characteristics, they can work together to provide frequency regulation services for the power system under unified dispatch. Furthermore, this paper focuses primarily on downward regulation services in secondary frequency regulation [23].

#### 2.2.1. Distributed Energy Storage

Distributed energy storage systems (ESS) can continuously adjust their charging and discharging power, making them key resources for VPPs participating in secondary frequency regulation. For energy storage unit *i*, its regulation capacity in AGC downward regulation services is primarily constrained by its rated charging power, available reserve capacity, and energy storage state. The power and energy constraints for an energy storage unit can be expressed as [4,5]:

(1) $$\sum P_{i}^{\mathrm{AGC}}+\Delta b_{i}\leq {\bar{P}}_{i}^{\mathrm{ch}}$$

(2) $$E_{i}+\eta_{i}^{c}T\left(\sum P_{i}^{\mathrm{AGC}}+b_{i}^{c}\right)\leq {\bar{E}}_{i}$$

(3) $$E_{i}+\eta_{i}^{c}T{\left(\sum P_{i}^{\mathrm{AGC}}-b_{i}^{d}\right)}^{+}\leq {\bar{E}}_{i}$$

where $\Delta b_{i}=b_{i}^{c}-b_{i}^{d}$ denotes the net charging/discharging power of the energy storage system; $P_{i}^{\mathrm{AGC}}=P_{i(\mathrm{AGC},\mathrm{cin})}$ represents the AGC dispatch command power of unit *i*; $b_{i}^{c}$ and $b_{i}^{d}$ are the charging and discharging powers, respectively; $\eta_{i}^{c}$ is the charging efficiency; *T* is the duration of the dispatch interval; and ${\left(\cdot \right)}^{+}≜\max\left\{0,\cdot \right\}$ denotes the positive part operator. These constraints ensure that the energy storage system does not exceed the power and energy limits when participating in frequency regulation, thereby meeting the requirements for safe operation of the equipment [5].

#### 2.2.2. Load Control

Unlike energy storage DERs, controllable loads are incapable of providing reserve capacity or continuously modulating their output in response to frequency regulation signals [7]. Consequently, these devices are aggregated into a cluster and subjected to unified on/off control for frequency regulation execution. Upon receiving a regulation command, the load type DERs within the cluster coordinate to collectively deliver $P_{LD(\mathrm{AGC},\mathrm{dn})}$ units of reserve capacity. Given the inherently discrete nature of load characteristics, energy storage technology is employed to bridge the gap, transforming the stepped output of the load pool into a smooth, continuous signal. Should the load pool’s output prove insufficient to satisfy the downward regulation requirement, the energy storage system compensates by ramping up its charging power until the aggregate regulation power attains $-A_{t}\cdot P_{LD(\mathrm{AGC},\mathrm{dn})}$. For AGC services, the AGC command $A_{t}\in \left[-1,0\right]$ sent by the system operator is a continuous range with a value domain of $\left[-1,0\right]\times P_{LD(AGC,dn)}$.

Consequently, the AGC command for the load pool is bifurcated into two distinct signals, each corresponding to the reserve capacity provided by the energy storage system and the load pool, respectively. These signals exhibit a discrete ramp shaped profile with *S* steps on one flank. Following the formulation in Reference [5], the decomposed signals for the load pool and energy storage are given by:

(4) $$\sum_{i\in LD}p_{i,t(\mathrm{AGC},\mathrm{dn})}=-\phi \left(A_{t}\right)\times P_{LD(\mathrm{AGC},\mathrm{dn})}$$

(5) $$\sum_{i\in ES}P_{i(\mathrm{AGC},\mathrm{dn})}^{l}=\frac{P_{LD(\mathrm{AGC},\mathrm{dn})}}{S}$$

#### 2.2.3. Model of Communication Latency

VPP participation in AGC frequency regulation relies on communication networks to transmit dispatch instructions and relay resource status information. Many studies simplify communication links as ideal channels, neglecting the impact of factors such as bandwidth constraints, queue congestion, and insufficient communication reliability on frequency regulation control. However, in practical cellular wireless communication environments, communication latency directly affects the timeliness of AGC instruction execution, thereby impacting the frequency tracking performance of VPPs. In this paper, communication latency refers to the end to end delay of AGC dispatch instruction delivery along the control path from the control centre to the VPP management platform and then to DER units. Here, $t_{1}$ and $t_{2}$ denote the scheduling delay and processing delay at the VPP management platform, respectively, where regulation commands for DERs are generated and formatted for transmission. $t_{3}$ denotes the transmission delay from the VPP management platform to the corresponding base station. $t_{4}$ denotes the queueing delay at the base station, which depends on the traffic load of DER services and other coexisting services. $t_{5}$ denotes the delay associated with command delivery from the base station to DER units, including wireless transmission and terminal side processing delays [5]:

(6) $$t=t_{1}+t_{2}+t_{3}+t_{4}+t_{5}\;$$

According to URLLC latency studies, AGC commands are small control packets, and the physical transmission, propagation, and fixed backhaul delays are usually at the submillisecond to millisecond level. For example, the studies in [25,26] report millisecond level URLLC latency requirements and use a 0.1 ms frame duration and a 0.1 ms backhaul delay under the considered URLLC setting. Therefore, these relatively deterministic delay components are small compared with the overall AGC command delivery delay budget and can be incorporated into the delay threshold. Processing and scheduling delays at the VPP platform, BS, and DER terminals are mainly related to command generation, formatting, and device implementation, and are less sensitive to instantaneous traffic load. By contrast, the BS queueing delay varies with DER access intensity and coexisting service traffic, and may become the main stochastic contributor to delay violation when the packet arrival rate approaches the service rate. Therefore, this paper focuses on modeling the traffic dependent BS queueing delay $t_{4}$, while the other relatively stable delay components are absorbed into the delay threshold [4,25,26,27].

### 2.3. Construction of Energy Information Model

#### 2.3.1. Effective Capacity Communication Model

When VPPs participate in AGC frequency regulation services, they rely on communication networks to transmit AGC commands issued by the dispatch centre to distributed resource terminals in real time. Since wireless link capacity varies over time, AGC commands are subject to factors such as queueing, scheduling, and channel fluctuations during transmission, which may prevent them from being delivered within the current dispatch cycle. To characterize the support capability of communication networks for VPP frequency regulation services under statistical delay constraints, this paper introduces effective capacity (EC) theory to model the statistical service capability of 5G network slicing [28].

According to EC theory, a wireless link can be regarded as a random service process. Let $S_{n}\left(T\right)$ denote the random service volume provided by the *n*th slice to the VPP frequency regulation service within a time slot *T*. If the VPP subscribes to *N* slices, the aggregate service process can be expressed as

(7) $$S_{N}\left(T\right)=\sum_{n=1}^{N}S_{n}\left(T\right)\;$$

Given a QoS exponent θ>0, the effective capacity of the aggregate service process is defined as [28,29]

(8) $$EC_{N}\left(\theta \right)=-\frac{1}{\theta T}\ln E\left[e^{-\theta S_{N}\left(T\right)}\right]$$

where θ reflects the strictness of the statistical delay constraint. A larger θ corresponds to a more delay sensitive service. Since AGC commands are typical low latency and high reliability control messages, their transmission is subject to stringent statistical QoS constraints [4,25]. The effective capacity in (8) characterizes the statistical service capability of the sliced wireless link.

#### 2.3.2. Model of Delay Default Probability

Let λ denote the average arrival rate of AGC command traffic, and let $D_{\max}$ denote the maximum allowable delay threshold. According to large deviation based statistical QoS theory [28,30], when the queue is stable, i.e., $EC_{N}\left(\theta \right)>\lambda$, the delay violation probability can be approximated as

(9) $$\mathrm{Pr}\left\{D>D_{\max}\right\}\approx \exp\left[-\theta \left(EC_{N}\left(\theta \right)-\lambda \right)D_{\max}\right]\;$$

Equation (9) shows that the delay violation probability decreases exponentially with the effective service margin $EC_{N}\left(\theta \right)-\lambda$ and the delay threshold $D_{\max}$. As the number of subscribed slices *N* increases, $EC_{N}\left(\theta \right)$ increases, and the probability that an AGC command misses the current scheduling cycle decreases.

Since VPP frequency regulation depends on whether AGC commands arrive in time rather than whether they are eventually received, this paper defines the timely AGC command delivery probability as

(10) $$P_{s}\left(N\right)=1-\mathrm{Pr}\left\{D>D_{\max}\right\}\;$$

Substituting (9) into (10), we obtain

(11) $$P_{s}\left(N\right)=1-\exp\left[-\theta \left(EC_{N}\left(\theta \right)-\lambda \right)D_{\max}\right]\;$$

Equation (11) establishes the analytical relationship between the number of subscribed 5G slices *N* and the timely AGC command delivery probability $P_{s}\left(N\right)$, which provides the basis for mapping communication performance to the VPP frequency regulation performance score.

#### 2.3.3. Model for Evaluating Frequency Regulation Performance

Global system operators usually evaluate frequency regulation performance by comparing the AGC command sequence with the actual response sequence within a market settlement period. In this paper, the AGC tracking accuracy score is calculated as [4]

(12) $$AS_{AGC}=1-\frac{\sum_{k=1}^{K}\left|A_{k}-{\hat{A}}_{k}\right|}{\sum_{k=1}^{K}\left|A_{k}\right|}\;$$

where $A_{k}$ denotes the normalized AGC command at the *k*th control interval, ${\hat{A}}_{k}$ denotes the normalized actual response of the VPP, and *K* denotes the number of AGC control intervals in one settlement period.

Before evaluating the aggregate response, the VPP decomposes the system level AGC command $A_{k}$ into DER level commands according to its internal dispatch strategy. Let $A_{i,k}$ denote the AGC command assigned to the *i*th DER at the *k*th control interval. The normalized aggregate command corresponding to all DER level dispatch commands is expressed as

(13) $$A_{k}^{\mathrm{agg}}=\frac{\sum_{i}A_{i,k}}{P_{\mathrm{reg}}}$$

where $P_{\mathrm{reg}}$ denotes the committed regulation capacity used for response normalization.

Due to communication delay or packet loss, DERs may fail to receive the latest AGC command within the current control interval. In this case, the VPP response is modeled using a hold last command mechanism [4], i.e., if the latest command is not delivered on time, the resource continues to follow the most recently valid command. Let $P_{s}\left(N\right)$ denote the timely AGC command delivery probability when the VPP subscribes to *N* slices. Then, the expected normalized VPP response satisfies

(14) $${\bar{A}}_{k}=P_{s}\left(N\right)A_{k}^{\mathrm{agg}}+\left[1-P_{s}\left(N\right)\right]{\bar{A}}_{k-1}\;$$

where ${\bar{A}}_{k}=E\left[{\hat{A}}_{k}\right]$ denotes the expected normalized response at the *k*th control interval.

Equivalently, the recursive expression can be expanded as

(15) $${\bar{A}}_{k}=\sum_{j=0}^{k-1}P_{s}\left(N\right){\left[1-P_{s}\left(N\right)\right]}^{j}A_{k-j}^{\mathrm{agg}}+{\left[1-P_{s}\left(N\right)\right]}^{k}{\bar{A}}_{0}\;$$

Equation (15) shows that the current response may correspond to the current AGC command or to a historical command. When several consecutive command delivery failures occur, the VPP response is increasingly affected by previously received valid commands.

In the numerical calculation, the expected response sequence ${\bar{A}}_{k}$ is used as a surrogate for the actual response sequence ${\hat{A}}_{k}$ in (12). Therefore, the relationship between 5G slice subscription and frequency regulation performance can be summarized as

(16) $$N\to EC_{N}\left(\theta \right)\to P_{s}\left(N\right)\to {\bar{A}}_{k}\to AS_{AGC}\;$$

This mapping chain connects communication resource subscription, delay constrained AGC command delivery, VPP response tracking, and frequency regulation performance scoring, providing the basis for constructing the Stackelberg game utility functions of the VPP and the communication service provider.

## 3. Formulation and Solution of the Stackelberg Game

When VPPs participate in frequency regulation services, the reliability of AGC command transmission directly affects their regulation performance score and market revenue. To improve communication reliability, the VPP can subscribe to 5G network slices from the communication service provider. However, subscribing to more slices increases the VPP’s communication cost, while the communication service provider must balance slice revenue and resource provisioning cost. Therefore, the interaction between the VPP and the communication service provider involves both operational coupling and conflicting economic interests.

In this paper, the communication service provider has the pricing authority for network slicing resources, while the VPP determines the number of subscribed slices according to the announced slice price. This leader follower decision process is modeled as a Stackelberg game [31]. Specifically, the communication service provider acts as the leader and sets the unit slice price first, while the VPP acts as the follower and determines the optimal slice subscription quantity to maximize its own net revenue.

### 3.1. Stackelberg Game Model

#### 3.1.1. Follower: VPP Optimization Model

During a market settlement period, the VPP obtains revenue by providing AGC frequency regulation services and incurs communication costs by subscribing to 5G network slices. Let *N* denote the number of slices subscribed by the VPP, and let μ denote the unit slice price. The VPP’s net utility is expressed as

(17) $$U_{\mathrm{VPP}}\left(N,\mu \right)=B\left(N\right)-\mu N\;$$

where

(18) $$B\left(N\right)=p_{a}P_{\mathrm{reg}}AS\left(N\right)$$

denotes the gross regulation benefit of the VPP [32]. Here, $p_{a}$ is the unit frequency regulation service price, $P_{\mathrm{reg}}$ is the regulation capacity provided by the VPP, and AS(N) is the AGC performance score obtained under the slice subscription quantity *N*. The term μN represents the communication cost paid by the VPP to the communication service provider.

For a given slice price μ, the VPP determines its optimal slice subscription quantity by solving

(19) $$\begin{array}{cc} \max_{N}\; & U_{\mathrm{VPP}}\left(N,\mu \right)\\ s.t.\; & N\in N=\{0,1,\ldots ,N_{\max}\} \end{array}$$

Since N is a finite discrete set, the VPP’s best response can be obtained by enumerating all feasible slice quantities:

(20) $$N^{*}\left(\mu \right)=\arg\max_{N\in N}U_{\mathrm{VPP}}\left(N,\mu \right)\;$$

#### 3.1.2. Leader: Communication Service Provider Optimization Model

The communication service provider obtains revenue by selling network slices to the VPP and incurs resource provisioning costs. Its utility function is given by

(21) $$U_{\mathrm{op}}\left(\mu ,N\right)=\mu N-C\left(N\right)\;$$

where C(N) denotes the cost of providing *N* slices. Considering that additional slices may introduce bandwidth occupation, resource scheduling overhead, and congestion related management costs, the cost function is modeled as a convex function:

(22) $$C\left(N\right)=c_{1}N+c_{2}N^{2}\;$$

where $c_{1}$ denotes the linear resource cost and $c_{2}$ denotes the additional marginal cost caused by congestion and resource management.

Anticipating the VPP’s best response $N^{*}\left(\mu \right)$, the communication service provider determines its optimal slice price by solving

(23) $$\begin{array}{cc} \max_{\mu }\; & U_{\mathrm{op}}\left(\mu ,N^{*}\left(\mu \right)\right)\\ s.t.\; & \mu \in M=[\mu_{\min},\mu_{\max}] \end{array}\;$$

Thus, the optimal pricing strategy is

(24) $$\mu^{SE}=\arg\max_{\mu \in M}U_{\mathrm{op}}\left(\mu ,N^{*}\left(\mu \right)\right)$$

and the corresponding slice subscription quantity is

(25) $$N^{SE}=N^{*}\left(\mu^{SE}\right)$$

### 3.2. Stackelberg Equilibrium

**Definition** **1** (Stackelberg Equilibrium [31])**.** *The strategy pair $\left(\mu^{SE},N^{SE}\right)$ is a Stackelberg equilibrium if the following conditions hold:*

(26) $$\begin{array}{c} U_{\mathrm{VPP}}\left(N^{SE},\mu^{SE}\right)\geq U_{\mathrm{VPP}}\left(N,\mu^{SE}\right),\;\forall N\in N\; \end{array}$$

and

(27) $$\begin{array}{c} U_{\mathrm{op}}\left(\mu^{SE},N^{*}\left(\mu^{SE}\right)\right)\geq U_{\mathrm{op}}\left(\mu ,N^{*}\left(\mu \right)\right),\;\forall \mu \in M \end{array}$$

Since the VPP’s feasible strategy set N is finite, the VPP’s best response exists for any given μ. The communication service provider’s feasible price set M is closed and bounded. Therefore, the upper level optimization problem can be solved by evaluating $U_{\mathrm{op}}\left(\mu ,N^{*}\left(\mu \right)\right)$ over the feasible price interval. In this paper, a one dimensional search method is used to obtain $\mu^{SE}$, and the corresponding $N^{SE}$ is obtained through the VPP’s best response.

### 3.3. Stability Analysis of the Cooperative Benchmark

To further clarify the equilibrium properties of the proposed decision framework, this paper introduces an aggregate welfare benchmark as a reference case for system level efficiency and defines unilateral deviation gain indicators for both participants. These indicators are used to examine the incentive consistency of the benchmark decision and to provide an additional economic interpretation of the interaction between the VPP and the communication service provider [33,34].

The cooperative welfare maximizing slice allocation is defined as [35,36]

(28) $$N^{c}=\arg\max_{N\in N}W\left(N\right)\;W\left(N\right)=B\left(N\right)-C\left(N\right)\;$$

where W(N) denotes the aggregate welfare of the VPP and the communication service provider. The slice payment μN is an internal transfer between the two participants and, therefore, cancels out in the aggregate welfare calculation [35,36].

Let $\left(\mu^{c},N^{c}\right)$ denote the cooperative benchmark solution, where $N^{c}$ is determined by the welfare maximizing slice allocation in (28). To examine whether this cooperative solution is self enforcing, the unilateral deviation gain of the VPP is defined as [34,37,38]

(29) $$G_{\mathrm{VPP}}^{c}=\max_{N\in N}U_{\mathrm{VPP}}\left(N,\mu^{c}\right)-U_{\mathrm{VPP}}\left(N^{c},\mu^{c}\right)\;$$

Equation (29) measures whether the VPP can improve its own utility by changing the slice subscription quantity while the slice price remains at the cooperative level $\mu^{c}$. If $G_{\mathrm{VPP}}^{c}>0$, the VPP has an incentive to deviate from the cooperative slice allocation.

Similarly, the unilateral deviation gain of the communication service operator is defined as

(30) $$G_{\mathrm{op}}^{c}=\max_{\mu \in M}U_{\mathrm{op}}\left(\mu ,N^{*}\left(\mu \right)\right)-U_{\mathrm{op}}\left(\mu^{c},N^{c}\right)$$

Equation (30) measures whether the communication service provider can improve its own utility by abandoning the cooperative price and returning to its individual pricing decision, while anticipating the VPP’s best response $N^{*}\left(\mu \right)$. If $G_{\mathrm{op}}^{c}>0$, the provider has an incentive to deviate from the cooperative solution.

Therefore, the cooperative benchmark is self enforcing only if [34,38]

(31) $$G_{\mathrm{VPP}}^{c}\leq 0,\;G_{\mathrm{op}}^{c}\leq 0\;$$

If $G_{\mathrm{VPP}}^{c}>0$ and $G_{\mathrm{op}}^{c}>0$, both participants can improve their individual utilities by deviating from the cooperative solution. In this case, although the cooperative benchmark may achieve a higher total welfare, it is not self enforcing in the sense that both participants have incentives to leave the cooperative state. Therefore, additional coordination mechanisms, such as enforceable contracts, long-term agreements, or side payments, are required to sustain the cooperative outcome.

## 4. Simulation Analysis

### 4.1. Simulation Settings

To verify the effectiveness of the proposed method, this paper designs three typical scenarios for comparative analysis:

- COEC scenario: The Stackelberg game model based on EC theory proposed in this paper is adopted to collaboratively optimize the pricing and configuration of slicing resources.
- NCOEC scenario: The communication operator and VPP lack effective coordination and make decisions only according to their respective local interests.
- CONEC scenario: Both parties make collaborative decisions, but EC theory is not introduced to characterize communication performance.

This section verifies the effectiveness of the proposed hierarchical decision making mechanism through numerical simulations. For the VPP, the maximum number of 5G network slices that can be purchased is set to $N_{\max}=120$ [39,40]. The AGC frequency regulation service price is set to $P_{a}=\$24/\mathrm{MW}$ [4], and the committed regulation capacity is set to $P_{\mathrm{reg}}=12\;\mathrm{MW}$. The maximum allowable communication latency is set to $D_{\max}=0.22$ s, the QoS sensitivity coefficient is set to θ=15, and the AGC service demand is set to $\lambda_{\mathrm{eq}}=0.50\;\mathrm{MW}$ [4,28,41,42].

The physical meanings of the main simulation parameters are summarized in Table 1.

The market related parameters follow PJM style regulation market settings, and their impacts on the equilibrium results are further evaluated through sensitivity analysis [4]. The communication and control performance indicators obtained under the three scenarios are summarized in Table 2. The comparison includes the AS, AGC command reception success probability, optimal slice subscription number, and total system utility. The adopted parameters’s differences from practical engineering applications are summarized in Table 3.

### 4.2. Economic Benefit Comparison

**Figure 3:**
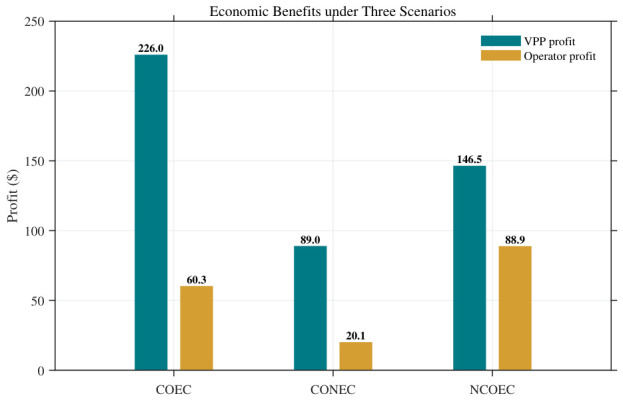
Economic benefit comparison under three scenarios.

Figure 3 compares the economic returns of the three scenarios. COEC gives the highest VPP profit of $226.0, which is 153.9% higher than CONEC and 54.3% higher than NCOEC. The operator profit in COEC is $60.3, which is 200.0% higher than that in CONEC. Although NCOEC yields a higher operator only profit, its VPP profit and total system utility are lower than those of COEC. Therefore, the EC based pricing rule improves the coordinated economic outcome of the VPP and the communication service provider.

### 4.3. Communication and Tracking Performance

**Figure 4:**
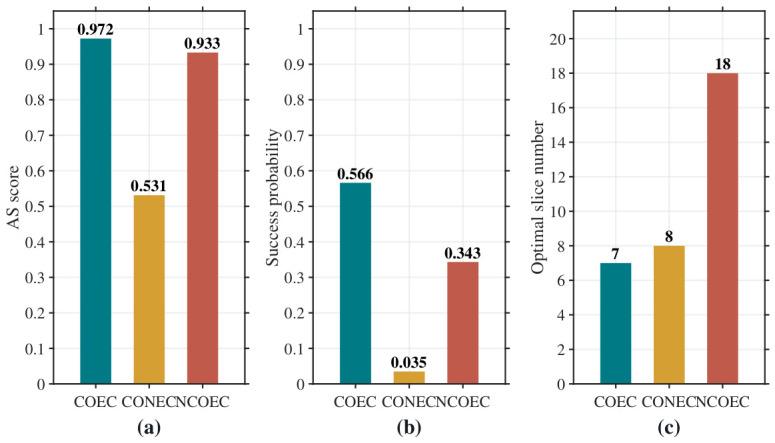
Communication and control performance comparison under three scenarios: (**a**) AGC tracking performance measured by AS scores; (**b**) communication reliability represented by success probabilities; (**c**) optimal slice subscription numbers.

**Figure 5:**
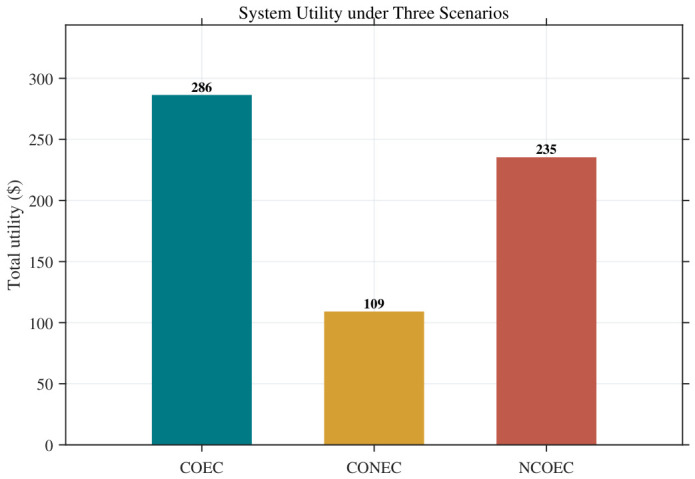
System total utility comparison under three scenarios.

Figure 4 shows the control and communication indicators at equilibrium. COEC obtains the highest AS value of 0.9725 and the highest AGC command reception probability of 0.5663. Compared with CONEC, COEC improves the success probability by a factor of 16.23. Compared with NCOEC, COEC improves the success probability by 65.0% while using fewer subscribed slices. Figure 5 gives the total utility of the three scenarios. COEC reaches $286.35, which is 162.4% higher than CONEC and 21.7% higher than NCOEC. This means that a high AS alone is not enough. The slice cost and operator revenue must also be coordinated. The quantitative improvements are summarized in Table 4.

**Figure 6:**
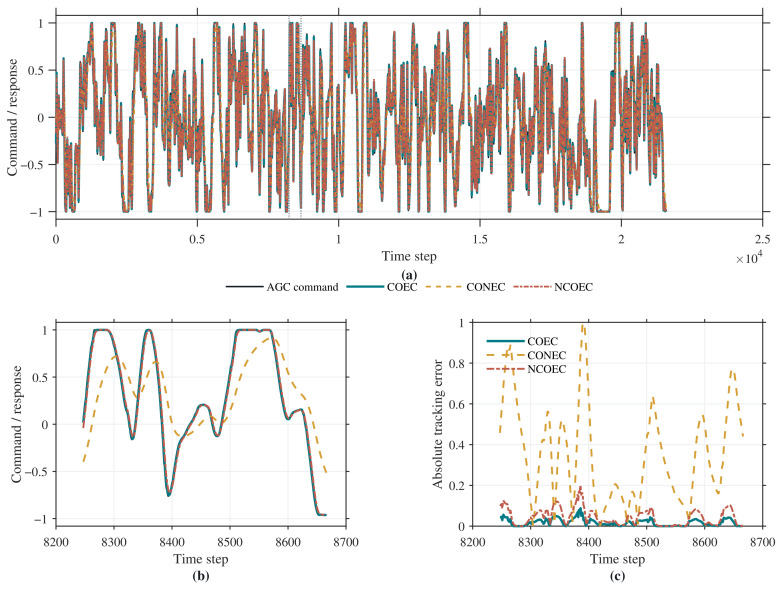
AGC tracking comparison under three scenarios: (**a**) full tracking horizon showing the AGC command and responses under the three scenarios. (**b**) Enlarged view of the local response over a selected time window. (**c**) Absolute tracking errors in the local window.

Figure 6 compares the AGC tracking trajectories. In the full horizon, the curves are dense and difficult to distinguish. The local zoom gives a clearer view of the response differences. COEC almost overlaps with the AGC command in the selected window, whereas CONEC shows visible delay and deviation. The local absolute error plot shows that COEC has the smallest tracking error in the displayed window. The numerical tracking performance is further quantified by the AS values reported in Table 4.

### 4.4. Slice Subscription and Equilibrium Formation

**Figure 7:**
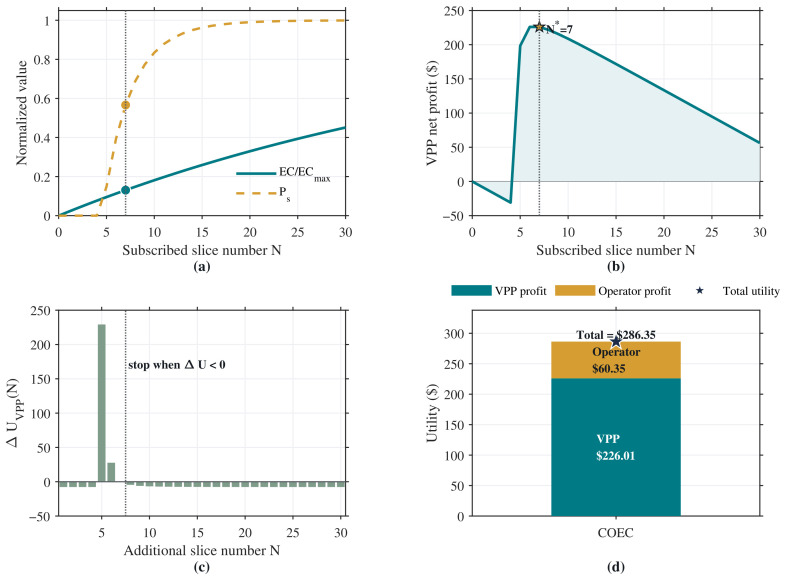
Economic equilibrium mechanism of slice subscription under the proposed COEC scheme: (**a**) communication performance growth with respect to the subscribed slice number, showing normalized EC and success probability $P_{s}$; (**b**) VPP net profit as a function of the slice number, indicating the follower optimum; (**c**) marginal net benefit of adding slices; (**d**) equilibrium profit decomposition between the VPP and the operator.

Figure 7 shows the communication performance which can improve as more slices are subscribed. However, the improvement becomes smaller after several slices. At the equilibrium price, the VPP net profit reaches its maximum at $N^{*}=7$. The marginal net benefit plot gives the same conclusion. After this point, an additional slice brings negative net benefit. Therefore, the equilibrium is an economic optimum.

**Figure 8:**
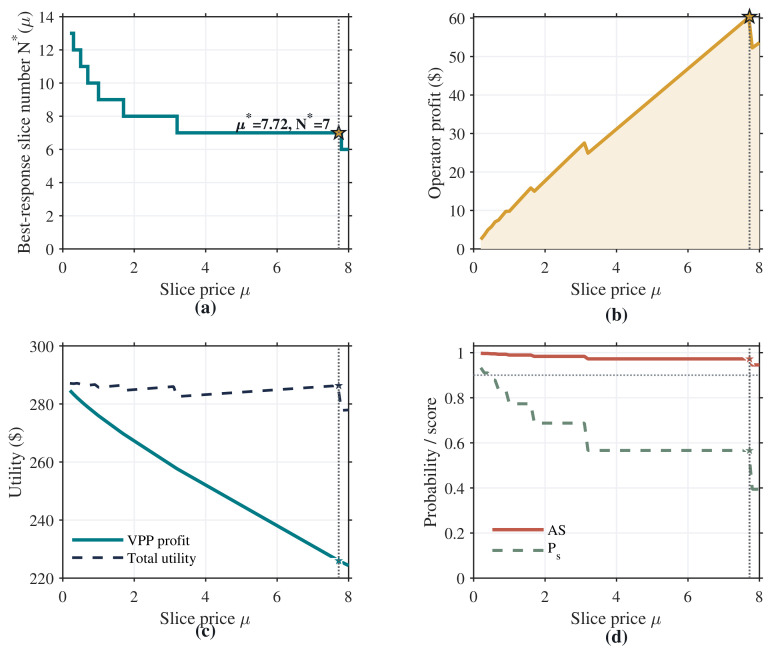
Stackelberg equilibrium formation under the proposed COEC scheme: (**a**) follower’s best response slice number $N^{*}$ versus the slice price μ; (**b**) leader’s objective (operator profit) versus the slice price μ; (**c**) follower payoff and total system utility versus the slice price μ; (**d**) communication and regulation performance metrics (AS and $P_{s}$) versus the slice price μ. In subfigures (**a**) and (**b**), the star marker denotes the Stackelberg equilibrium (SE) point and corresponds to the superscript SE used for the equilibrium solution in the text.

Figure 8 shows the Stackelberg equilibrium formation process. As the unit slice price increases, the VPP reduces its best response slice number. The operator profit first increases and then reaches its maximum at the marked point. Around this point, the VPP profit and total utility remain at acceptable levels, while the AS and $P_{s}$ curves remain stable. This confirms that the equilibrium price and subscription quantity are determined by the leader follower interaction between the operator and the VPP.

### 4.5. Robustness Case Studies

The slice size is scanned and the resulting slice value, slice cost, and net objective are compared, this subsection evaluates robustness through numerical parameter scans, each tested factor is varied while the remaining settings are kept at their baseline values, and the resulting AS, AGC command reception probability, VPP profit, operator profit, and total utility are recalculated.

**Figure 9:**
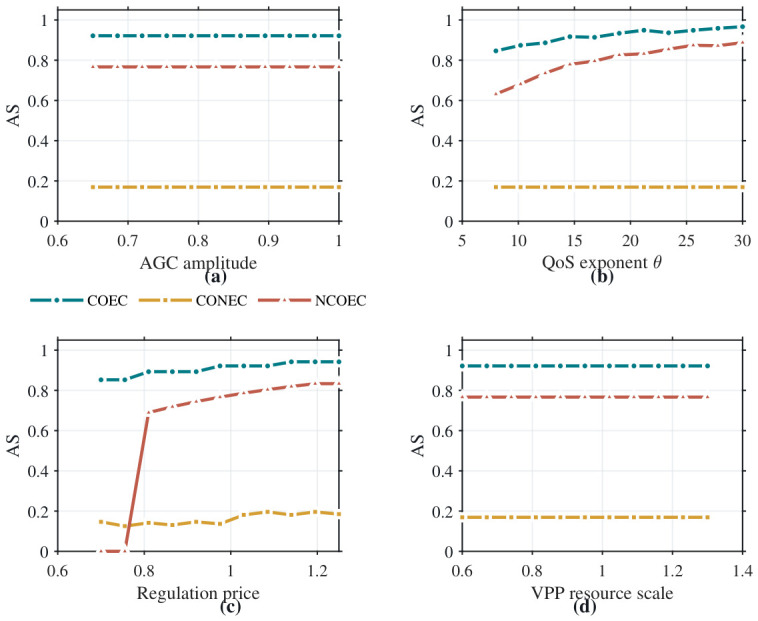
AS robustness comparison: (**a**) AGC amplitude scaling; (**b**) QoS exponent θ variation; (**c**) regulation price scaling; (**d**) VPP resource scale variation.

Figure 9 first reports AS robustness under four operating variations. The COEC curve remains near the top in all panels. CONEC remains much lower because it lacks the EC based reliability mapping. NCOEC performs better than CONEC, but it is still below COEC in most cases. This indicates that the proposed scheme is not effective only at one operating point; it also maintains stable tracking performance when the AGC amplitude, QoS exponent, regulation price, or VPP resource scale changes.

Following the same numerical scan logic, additional robustness checks are conducted for the key parameters that may affect the game equilibrium and benchmark comparison. The tested settings are summarized in Table 5. For scalar equilibrium related parameters, the value is varied around its baseline. For the slice price search interval, the interval width is varied while keeping the midpoint unchanged. For scenario specific coefficients, each coefficient is moved from the neutral value to its baseline value. Communication uncertainty is examined through network traffic load and an equivalent degradation factor for AGC command reception [43,44,45].

The robustness results are reported in Table 6. The minimum margin denotes the smallest total utility advantage of COEC over the best benchmark scenario among all sampled points. A positive margin means that COEC still achieves the highest total utility over the tested range.

As shown in Table 6, COEC maintains the highest total utility under all tested variations of the key equilibrium related parameters and scenario specific coefficients. Therefore, the main conclusion is not driven by a particular baseline parameter setting or by a particular choice of benchmark scaling coefficient.

Network traffic and unexpected communication degradation are further examined to address practical communication uncertainty [4,16,43]. For the network traffic case, the MW equivalent AGC service demand is increased from its baseline value toward the feasibility boundary implied by the effective capacity model. For the communication degradation case, an equivalent attenuation factor $\eta_{f}$ is applied to the AGC command reception probability, where $\eta_{f}=1$ denotes normal communication conditions and a smaller $\eta_{f}$ denotes stronger communication degradation.

As shown in Table 7, COEC maintains the highest total utility under both increased network traffic load and degraded communication reliability. These results further support the robustness of the proposed mechanism under communication uncertainty.

## 5. Conclusions

To address the issues of unclear mechanisms affecting communication reliability and the lack of pricing strategies for slice resources when 5G slicing based VPPs participate in frequency regulation, this study proposes a collaborative optimization method based on Stackelberg game theory. By modeling effective capacity and incorporating AS scores into the communication aware utility function, this method achieves the joint optimization of communication resource allocation, frequency regulation performance, and the economic benefits of both parties. The results indicate that the proposed method not only yields more efficient optimization solutions but also increases telecommunications operators revenue while optimizing the frequency regulation revenue of the VPP.

Future research will place greater emphasis on incorporating real world 5G network measurement data into the parameter calibration and validation process, as well as investigating the computational complexity and scalability of the proposed framework in large scale VPP systems with massive DER aggregation and multi-base station communication environments. In addition, future work will further explore long-term coordination mechanisms between VPPs and communication service providers, including incentive compatible pricing, contractual arrangements, and adaptive resource subscription strategies under practical operational uncertainties.

## Figures and Tables

**Table 1:** Physical meaning of main simulation parameters.

Symbol	Value	Unit	Physical Meaning
$N_{\max}$	120	–	Maximum number of purchasable slices
$P_{a}$	24	$/MW	AGC regulation service price
$P_{\mathrm{reg}}$	12	MW	Committed regulation capacity
$D_{\max}$	0.22	s	Maximum allowable AGC command latency
θ	15	–	Sensitivity to delay violation risk
$\lambda_{\mathrm{eq}}$	0.50	MW	MW equivalent AGC service demand
$E_{\max}^{\mathrm{eq}}$	5.75	MW	Maximum MW equivalent effective capacity
*k*	0.020	–	Growth coefficient of effective capacity

**Table 2:** Performance Comparison of Communication and Control Indicators.

Indicators	AS	Success Probability	Optimal *N*	Total Utility ($)
COEC	0.9725	0.5663	7	286.35
CONEC	0.5312	0.0349	8	109.14
NCOEC	0.9330	0.3433	18	235.37

**Table 3:** Differences from practical engineering applications.

Parameter Type	Practical Engineering Difference
Market parameters	Vary with market clearing results and bidding strategies
Regulation capacity	Depends on DER availability and VPP dispatch capability
Slice number	Limited by operator resource pools and SLA configurations
Communication latency	Should be measured from real 5G network operation
Success probability	Should be estimated from packet delivery and delay data
MW equivalent parameters	Require calibration using field measurements
Scenario specific coefficients	Require calibration according to practical operating scenarios

**Table 4:** Quantitative improvement of COEC over benchmark scenarios.

Metric	COEC Value	Gain over CONEC	Gain over NCOEC
AS score	0.9725	83.1%	4.2%
Success probability $P_{s}$	0.5663	16.23 times	65.0%
VPP profit ($)	226.0	153.9%	54.3%
Operator profit ($)	60.3	200.0%	−32.2%
Total utility ($)	286.35	162.4%	21.7%

**Table 5:** Numerical scan settings for robustness case studies.

Case Study Item	Tested Factor	Variation Setting
Equilibrium related parameters	$c_{1}$, $c_{2}$, $E_{\max}^{\mathrm{eq}}$, *k*, $D_{\max}$	One factor perturbation around baseline
Slice price search	$\left[\mu_{\min},\mu_{\max}\right]$	Search range perturbation
Scenario coefficients	$\gamma_{\mathrm{op}}$, $\gamma_{\mathrm{cost}}$, $\gamma_{\mathrm{rev}}$, $\gamma_{\mathrm{EC}}$	Neutral to baseline scan
Communication uncertainty	Traffic load and degradation factor	Stress test scan

**Table 6:** Robustness results for COEC total utility dominance.

Tested Factor	COEC Best Total Utility	Minimum Margin
Operator linear cost coefficient $c_{1}$	Yes	70.74
Operator quadratic cost coefficient $c_{2}$	Yes	70.70
Maximum effective capacity $E_{\max}^{\mathrm{eq}}$	Yes	65.97
Effective capacity fitting coefficient *k*	Yes	68.11
Maximum allowable latency $D_{\max}$	Yes	64.81
Slice price search range $\left[\mu_{\min},\mu_{\max}\right]$	Yes	69.92
COEC operator revenue coefficient	Yes	61.25
CONEC operator revenue coefficient	Yes	70.85
CONEC operator cost coefficient	Yes	70.85
NCOEC operator cost coefficient	Yes	70.30
NCOEC VPP revenue coefficient	Yes	42.71
NCOEC effective capacity coefficient	Yes	36.73

**Table 7:** Stress test results under communication uncertainty.

Tested Condition	Tested Range	COEC Best Total Utility
Network traffic load	Baseline to feasibility boundary	Yes
Communication degradation	Normal to severe degradation	Yes

## Data Availability

Data are contained within the article. No additional data are available.

## Nomenclatures

*N*	Number of subscribed 5G network slices
$N_{\max}$	Maximum number of purchasable 5G network slices
μ	Unit price of a network slice
$S_{n}\left(T\right)$	Service volume of the *n*th slice within time slot *T*
$S_{N}\left(T\right)$	Total service process provided by *N* subscribed slices
$EC_{N}\left(\theta \right)$	Effective capacity supported by *N* subscribed slices
$E_{N}^{\mathrm{eq}}$	MW equivalent effective capacity supported by *N* slices
θ	QoS sensitivity coefficient related to statistical delay violation risk
λ	Average arrival rate of AGC command traffic
$\lambda_{\mathrm{eq}}$	MW equivalent AGC service demand
*D*	Communication delay of AGC command transmission
$D_{\max}$	Maximum allowable AGC command delay
$P_{s}\left(N\right)$	Timely AGC command reception probability under *N* subscribed slices
$A_{k}$	Normalized AGC command at the *k*th control interval
${\hat{A}}_{k}$	Normalized actual response of the VPP
${\bar{A}}_{k}$	Expected normalized aggregate response of the VPP
$AS_{AGC}$	AGC performance score for frequency regulation tracking accuracy
$P_{a}$	AGC frequency regulation service price
$P_{\mathrm{reg}}$	Committed regulation capacity of the VPP
$U_{\mathrm{VPP}}$	Utility function of the VPP
$U_{\mathrm{op}}$	Utility function of the communication service provider
*W*	Aggregate welfare or total utility of the two participants
$c_{1}$, $c_{2}$	Linear and quadratic coefficients of the operator resource provisioning cost
$\gamma_{\mathrm{op}}$	Operator revenue scaling coefficient
$\gamma_{\mathrm{cost}}$	Operator cost scaling coefficient
$\gamma_{\mathrm{rev}}$	VPP revenue scaling coefficient
$\gamma_{\mathrm{EC}}$	Effective capacity scaling coefficient
$\rho_{\lambda }$	Network traffic load ratio
$\eta_{f}$	Communication degradation factor
ξ, ω	Perturbation factors used in sensitivity analysis
$\Delta W_{\min}$	Minimum total utility advantage of COEC over benchmark scenarios
$G_{\mathrm{VPP}}^{c}$	Unilateral deviation gain of the VPP from the benchmark decision
$G_{\mathrm{op}}^{c}$	Unilateral deviation gain of the communication service provider
VPP	Virtual power plant
DER	Distributed energy resource
AGC	Automatic generation control
EC	Effective capacity
BS	Base station
QoS	Quality of service
COEC	Cooperative scenario with effective capacity modeling
NCOEC	Noncooperative scenario with effective capacity modeling
CONEC	Cooperative scenario without effective capacity modeling
PJM	Pennsylvania-New Jersey-Maryland Interconnection
